# A novel fibrillin-1 gene missense mutation associated with neonatal Marfan syndrome: a case report and review of the mutation spectrum

**DOI:** 10.1186/s12887-016-0598-6

**Published:** 2016-04-30

**Authors:** Qian Peng, Yan Deng, Yuan Yang, Hanmin Liu

**Affiliations:** Department of Pediatric Cardiology, West China Second University Hospital/West China Women’s and Children’s Hospital, West China School of Medicine, Sichuan University, Chengdu, 610041 China; Key Laboratory of Birth Defects and Related Diseases of Women and Children (Sichuan University), Ministry of Education, Chengdu, 610041 China; Department of Pediatrics, Sichuan Academy of Medical Sciences & Sichuan Provincial People’s Hospital, Chengdu, 610072 China; Department of Cardiovascular Ultrasound and Non-invasive Cardiology, Sichuan Academy of Medical Sciences & Sichuan Provincial People’s Hospital, Chengdu, 610072 China; Department of Medical Genetics, West China Hospital, West China School of Medicine, Sichuan University, Chengdu, 610041 China

**Keywords:** Calcium-binding EGF-like domain, Cysteine substitution, Disulfide bond, *FBN1*, Neonatal Marfan syndrome

## Abstract

**Background:**

Marfan syndrome (MFS) is a heritable disorder of connective tissue resulting from pathogenic variants of the fibrillin-1 gene (*FBN1*). Neonatal Marfan syndrome (nMFS) is rare and the most severe form of MFS, involving rapidly progressive cardiovascular dysfunction leading to death during early childhood. The constant enrichment of the nMFS mutation spectrum is helpful to improve our understanding of genotype–phenotype correlations in the disease. Herein, we report a novel dominant mutation in exon 26 of *FBN1* (c.3331 T > C) in a sporadic case with nMFS.

**Case presentation:**

An 8-month-old Han Chinese girl presented with the classic nMFS phenotype, including prominent manifestations of bone overgrowth, aortic root dilatation, and multiple cardiac valve dysfunctions. Genetic analysis revealed that she was heterozygous for a *de novo FBN1* missense mutation (c.3331 T > C). The mutation leads to the substitution of a highly conserved FBN1 cysteine residue (p.Cys1111Arg), which is likely to severely perturb the FBN1 structure because of an alteration of the disulfide bond pattern in the calcium-binding epidermal growth factor-like (cbEGF) 12 domain. This variant was absent in 208 ethnically matched controls, providing further evidence that it may be causative of nMFS. An analysis of nMFS-associated mutations from the UMD-FBN1 database indicates that those *de novo* mutations altering disulfide bonds or Ca^2+^ binding sites of the cbEGF domains encoded by exons 25–33, and a lack of phenotypic heterogeneity may be associated with an increased risk for nMFS.

**Conclusion:**

We diagnosed an infant with rare nMFS showing rapidly progressive cardiovascular dysfunction and widely systemic features. As the only causal *FBN1* mutation identified in the patient, the missense mutation c.3331 T > C (p.Cys1111Arg) was associated with the severe phenotype of MFS. However, the pathogenicity of the novel mutation needs further confirmation in other patients with nMFS. Our review of the prominent characteristics of nMFS mutations relative to classic or incomplete MFS-related mutations will be helpful for the recognition of novel nMFS*-*associated variants.

**Electronic supplementary material:**

The online version of this article (doi:10.1186/s12887-016-0598-6) contains supplementary material, which is available to authorized users.

## Background

Marfan syndrome (MFS) (OMIM 154700) is an autosomal dominant disorder of fibrous connective tissue involving the ocular, skeletal, and cardiovascular systems. MFS patients present with clinical variability, in which the rare neonatal Marfan syndrome (nMFS) has the most severe presentation in early childhood [1]. The prognosis of nMFS is very poor, with a mean survival age of only 16.3 months [2]. Valvular insufficiencies and diaphragmatic hernias have been associated with shorter survival in patients diagnosed before the age of 1 year [3].

nMFS has been correlated with a limited number of mutations in the neonatal region of the fibrillin-1 gene (*FBN1*) (OMIM 134797) [4–6]. In the UMD-FBN1 mutations database (http://www.umd.be/), a total of 1,318 different *FBN1* mutations for MFS have been included to date, of which only 59 (4.8 %) including 37 missense mutations (2.8 %) are associated with nMFS. Here, we present a novel missense mutation associated with nMFS, which leads to a cysteine substitution in the calcium-binding epidermal growth factor-like (cbEGF) 12 domain of FBN1.

## Case presentation

An 8-month-old Han Chinese girl, the only child of her parents, was born full-term weighing 2.60 kg by vaginal delivery. Her father was 32 years old and her mother 26 years old. She was diagnosed with suspected MFS at birth caused by the presence of finger and toe arachnodactyly, elbow and knee flexion contractures, a characteristic ‘senile’ facial appearance, and loose skin, so was referred to the pediatrician. At the age of 8 days, an x-ray of the bilateral knee and elbow joints showed bone overgrowth with no apparent abnormalities of the joints or bone substance. After that, she received continuous follow-up at the Division of Children’s Healthcare, West China Second University Hospital, Chengdu, China. During this period, she was shown to have pectus excavatum, malnutrition, growth retardation, and feeding difficulties, with vitamin D levels at the lower end of normal limits; she was therefore administered vitamin D3 and calcium, although she failed to respond to treatment.

**Fig. 1:**
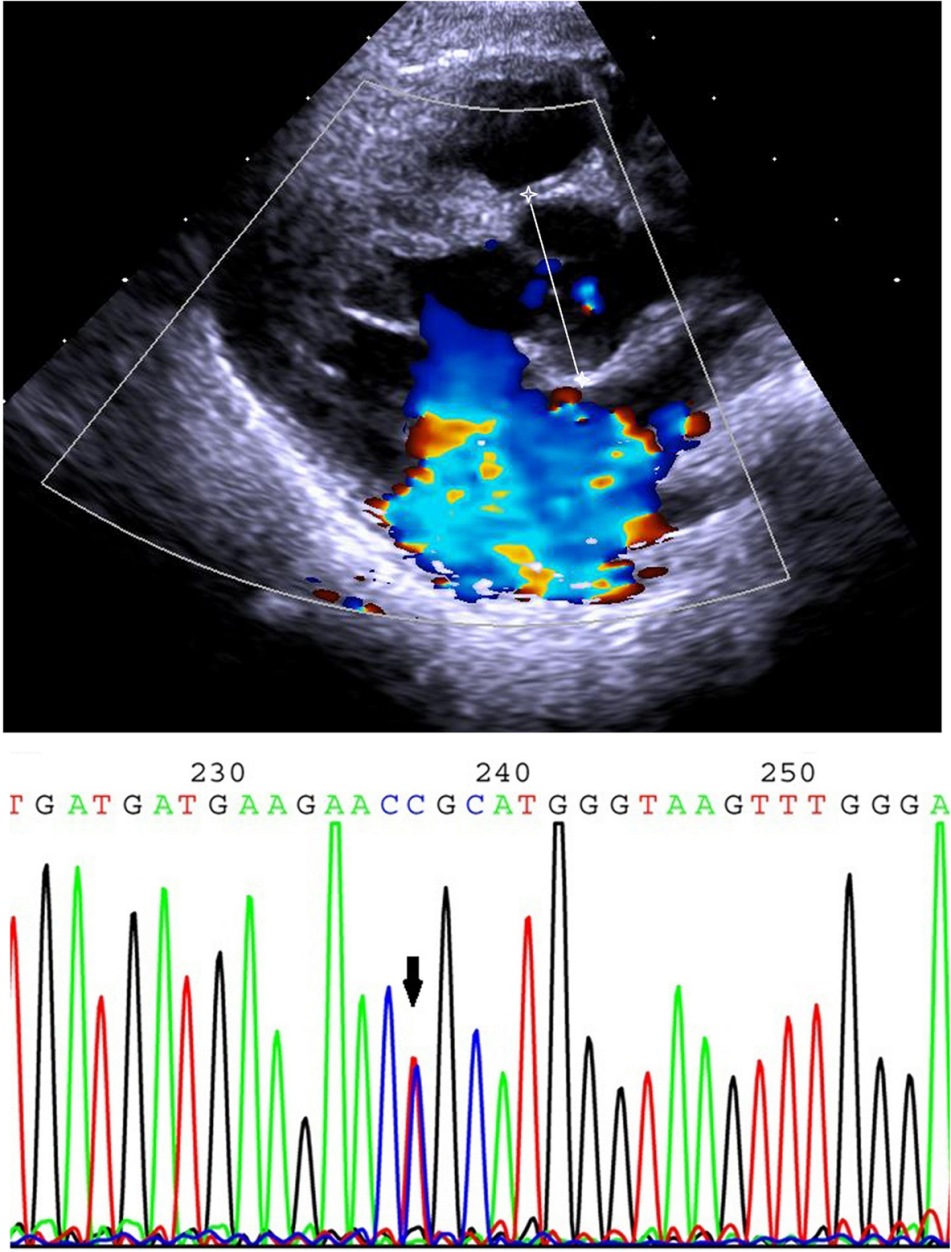
Echocardiograph showing aortic root dilatation at the sinuses of Valsalva (line in-between two asterisks) and the sequencing result showing the heterozygous missense mutation c.3331 T > C in the patient (arrow)

At the age of 6 months, she was admitted to hospital with bronchial pneumonia and then transferred to a pediatric cardiovascular ward because of a newly found grade 2–3 precordium murmur. On physical examination, in addition to malnutrition, she was observed to have a ‘senile’ facial appearance, loose skin, downslanting palpebral fissures, frontal bossing, downturned corners of the mouth, and skeletal abnormalities including big ears, micrognathia, arachnodactyly, a pectus deformity, pes planus, and dolichocephaly (Additional file 1). X-ray analysis of her bilateral calves showed that the margins of the distal tibial and fibular metaphysis were not smooth, and that the gap around the ankle was blurred. Cardiac enlargement and pectus excavatum were confirmed by computed tomography. Electrocardiography (ECG) suggested a sinus rhythm with abnormal Q waves in I and aVL leads. Echocardiography indicated mitral valve prolapse and regurgitation with a grade 3 insufficiency, tricuspid valve hypertrophy and regurgitation with a grade 1 insufficiency, left atrial chamber enlargement, and aortic root dilatation at the sinuses of Valsalva (23 mm, Z > 2) (Fig. 1). Ectopia lentis could not be determined because of the failure of pupils to dilate during several ophthalmologic examinations.

Neither parent had experienced any symptoms similar to those of their daughter, and their ECG examination and echocardiography were normal. According to the revised Ghent criteria for the diagnosis of MFS [7], the detected systemic features (scores) of the present patient included the wrist and thumb sign (3), pectus excavatum (1), pes planus (1), facial features (1), skin striae (1), and mitral valve prolapse (1). These systemic features (score >7) combined with the presentation of severe aortic root dilation (Z > 2) resulted in a diagnosis of MFS. After 5 days of antibiotic therapy administered intravenously for bronchial pneumonia, the patient recovered and was discharged. She was advised continuous follow-up at the Pediatric Cardiovascular Division to monitor cardiac function.

At the age of 8 months, the patient presented to the Department of Medical Genetics, West China Hospital, Chengdu, China. Her mother stated that no disease or condition had potentially affected the pregnancy, including hypertension, diabetes, thyroid disease, infection, medication, or toxic exposure. Moreover, the parents are not consanguineous and there is no family history of unexplained disorders or hereditary disease. After informed consent had been obtained, peripheral blood samples of the patient and her parents were collected for genetic testing to identify the causal *FBN1* mutation. All 65 exons of *FBN1* and their splice sites in the patient were sequenced by Sanger sequencing. This identified four variants, including a homozygous synonymous variant in exon 15 (c.1875 T > C, p.Asn625Asn) (rs25458), two heterozygous missense variants in exon 26 (c.3331 T > C, p.Cys1111Arg) (Fig. 1) and exon 27 (c.3442C > G, p.Pro1148Ala) (rs140598), and a heterozygous intronic variant (c.3464-5G > A) (rs11853943).

Total RNA was extracted from buccal epithelial cells and Sanger sequencing of the reverse transcriptase (RT)-PCR product further confirmed the presence of the variant c.3331 T > C. Direct sequencing of parental PCR products showed that substitution c.3331 T > C was absent in both parents, and also from 208 ethnically matched controls without the MFS phenotype. There was no evidence of parental mosaicism of the missense mutation. The patient’s mother was found to be homozygous for the c.3442C > G substitution. At this time, an echocardiogram of the patient showed a progression of mitral valve regurgitation with a grade 3–4 insufficiency. She was advised of the possibility of cardiovascular surgery if the severe mitral valve insufficiency led to further ventricular dysfunction. The CARE Checklist of information of the case report is available as Additional file 2.

## Discussion

The term neonatal Marfan syndrome was first used in 1991 to describe the most severe phenotype of MFS similar to cases previously known as infantile Marfan syndrome, congenital Marfan syndrome, and severe perinatal Marfan syndrome [1, 8–10]. Recently, it has been suggested that the term neonatal MFS should be replaced by early onset and rapidly progressive MFS to represent the most severe features of MFS in early childhood [11]. Of the 2,088 MFS patients on the UMD-FBN1 mutations database (last update, 28/08/14), only 80 (3.8 %) were recorded as suffering from nMFS, indicating that nMFS is a rare condition relative to classic and incomplete MFS. Its incidence rate is therefore far lower than that estimated for MFS, at 1/5,000–1/10,000 [11]. Although the characteristics of nMFS have been previously discussed [6, 12], there are currently no diagnostic criteria. In combination with systemic manifestations, the identification of *FBN1* mutations responsible for nMFS is helpful for disease diagnosis in the absence of any family history [7].

Our patient carries a *de novo* variant of *FBN1*, c.3331 T > C, which has not been reported previously. This missense substitution affects a cysteine residue in the cbEGF 12 domain (p.Cys1111Arg) of FBN1. Moreover, its absence in more than 200 ethnically matched controls suggested that it is a causative mutation [7]. It is of note that there is another missense mutation in the same codon (c.3332G > A, p.Cys1111Tyr) in the UMD-FBN1 mutations database leading to incomplete MFS. Although phenotypic variation of different mutations in the same codon has been observed in other codons encoding the disulfide bond-related cysteine residue of the cbEGF domain, the phenotypic consequence of the novel mutation in our patient needs further confirmation in other patients with nMFS. The nMFS diagnosis of our patient is supported by the high-degree similarity of clinical features to those reported previously [5, 13].

Most previously identified nMFS-associated *FBN1* mutations are known to cluster between exons 24 and 32, which is the neonatal region of *FBN1* [3, 4, 11, 12]. A recent hypothesis to explain this is that some mutations in the region may cause enhanced proteolytic susceptibility of FBN1 and loss of function for heparin binding, leading to a more severe phenotype relative to other mutations responsible for milder forms of MFS [14]. However, it is still difficult to predict the correlations between a given mutation in the region and the nMFS phenotype [11]. In recent years, more nMFS-causative mutations have been identified which may offer clues for the recognition of others.

**Fig. 2:**
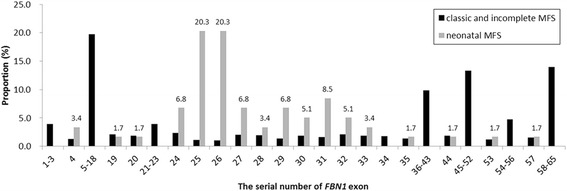
Different distributions of neonatal and classic or incomplete Marfan syndrome-associated mutations among *FBN1* exons based on the UMD-FBN1 mutations database

**Fig. 3:**
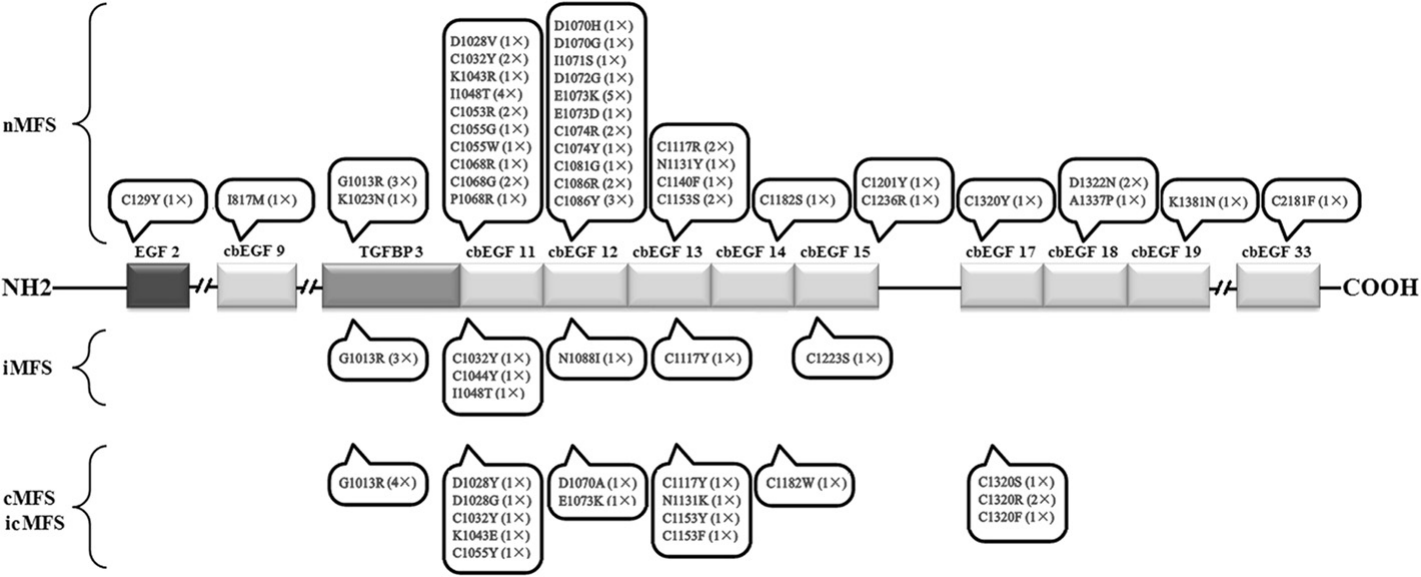
The location and phenotypic heterogeneity of amino acid substitutions in the FBN1 protein associated with neonatal Marfan syndrome, and the number of such substitutions in patients with MFS based on the UMD-FBN1 mutations database. nMFS, neonatal Marfan syndrome; iMFS, infantile Marfan syndrome; cMFS, classic Marfan syndrome; icMFS, incomplete Marfan syndrome

Based on information from the UMD-FBN1 mutations database, we have determined a number of characteristics of nMFS-associated mutations compared with those of classic and incomplete MFS. First, 92.3 % (60/65) of nMFS mutations were *de novo*, which is significantly higher than the number of *de novo* classic and incomplete MFS mutations (35.3 %, 417/1,181) (Fisher’s exact test, α = 0.05; *P* < 0.001). Second, at the genome level, the distribution of the two types of mutations differs among *FBN1* exons (Pearson’s χ test, α = 0.05; *P* < 0.001); in particular, most nMFS mutations (86.4 %, 51/59) cluster within exons 24–33 while the distribution of mutations for classic and incomplete MFS is more even with only 17.4 % (221/1274) in the exon 24–33 region (Fisher’s exact test, α = 0.05; *P* < 0.001) (Fig. 2). Third, at the protein level, 91.5 % (54/59) of nMFS mutations are located in cbEGF domains, which is significantly higher than that of mutations for classic and incomplete MFS (71.7 %, 914/1,274) (Fisher’s exact test, α = 0.05; *P* < 0.001). Within the domain cluster of cbEGFs 11–19 encoded by exons 25–33, 47 nMFS mutations are located, of which 43 (91.5 %) affect the disulfide bond or Ca^2+^ binding site. This compares with only 58.2 % (111/191) of classic and incomplete MFS mutations (Fisher’s exact test, α = 0.05; *P* < 0.001). An nMFS genotype–phenotype analysis showed that most of the mutations (88.4 %, 52/59) present exclusively in patients with nMFS. Further, of all missense mutations associated with nMFS, only 8.1 % (3/37) also present in patients with classic or incomplete MFS (Fig. 3). These observations strongly suggest that limited phenotypic heterogeneity of nMFS-associated mutations is evident, although it should not be ignored that some mutations can also result in a later onset or classic presentation of MFS.

A bicuspid aortic valve (BAV) is a common congenital heart abnormality [15] that appears to be associated with mutations in *FBN1* because of the significantly higher frequency of these mutations in affected patients relative to the general population [16, 17]. One of the *FBN1* variants in the current patient, c.3442C > G, has previously been reported to be a pathogenic mutation for BAV [18]. In the present study, the patient was a heterozygote and her mother a homozygote of the variant. However, echocardiography did not reveal BAV in either individual, which does not support causality of this variant for BAV.

## Conclusions

The diagnosis of the severe disease nMFS can be aided by identifying known nMFS-causing variants through continuous enrichment of the nMFS mutation spectrum. In the present study, we identified a novel dominant *FBN1* mutation, c.3331 T > C (p.Cys1111Arg), which was associated with the most severe phenotype of MFS. This finding will be helpful for the clinical diagnosis, prenatal diagnosis, and genetic counseling in patients with the same mutation. Our brief review, based on the latest database information, summarized the distinctive features of nMFS-associated mutations relative to mutations for classic and incomplete MFS, which will be valuable for evaluating the pathogenicity of novel *FBN1* variants for nMFS.

## Consent

Written informed consent was obtained from the patient’s parents for publication of this Case report including the results of genetic testing and any accompanying images. A copy of the written consent is available for review by the Editor of this journal.

## Ethics

This study was approved by the Ethics Committee of Clinical Trials and Biomedical Research, West China School of Medicine, Sichuan University, China.

## Additional files

Additional file 1: Clinical features of the patient showing facial appearance, dolichocephaly, the pectus deformity, arachnodactyly, the thumb sign, and pes planus. (JPEG 896 kb) Additional file 2: CARE Checklist (2013) of information to include when writing a case report. (DOCX 1484 kb)

## Abbreviations

BAV: bicuspid aortic valve
cbEGF: calcium-binding epidermal growth factor-like
ECG: electrocardiograph
*FBN1*: fibrillin-1
nMFS: neonatal marfan syndrome
