# Supplementary figures and images for: A novel fibrillin-1 gene missense mutation associated with neonatal Marfan syndrome: a case report and review of the mutation spectrum
Source: BMC Pediatr. 2016 Apr 30;16:60. doi: 10.1186/s12887-016-0598-6 (PMC4852411; doi:10.1186/s12887-016-0598-6)

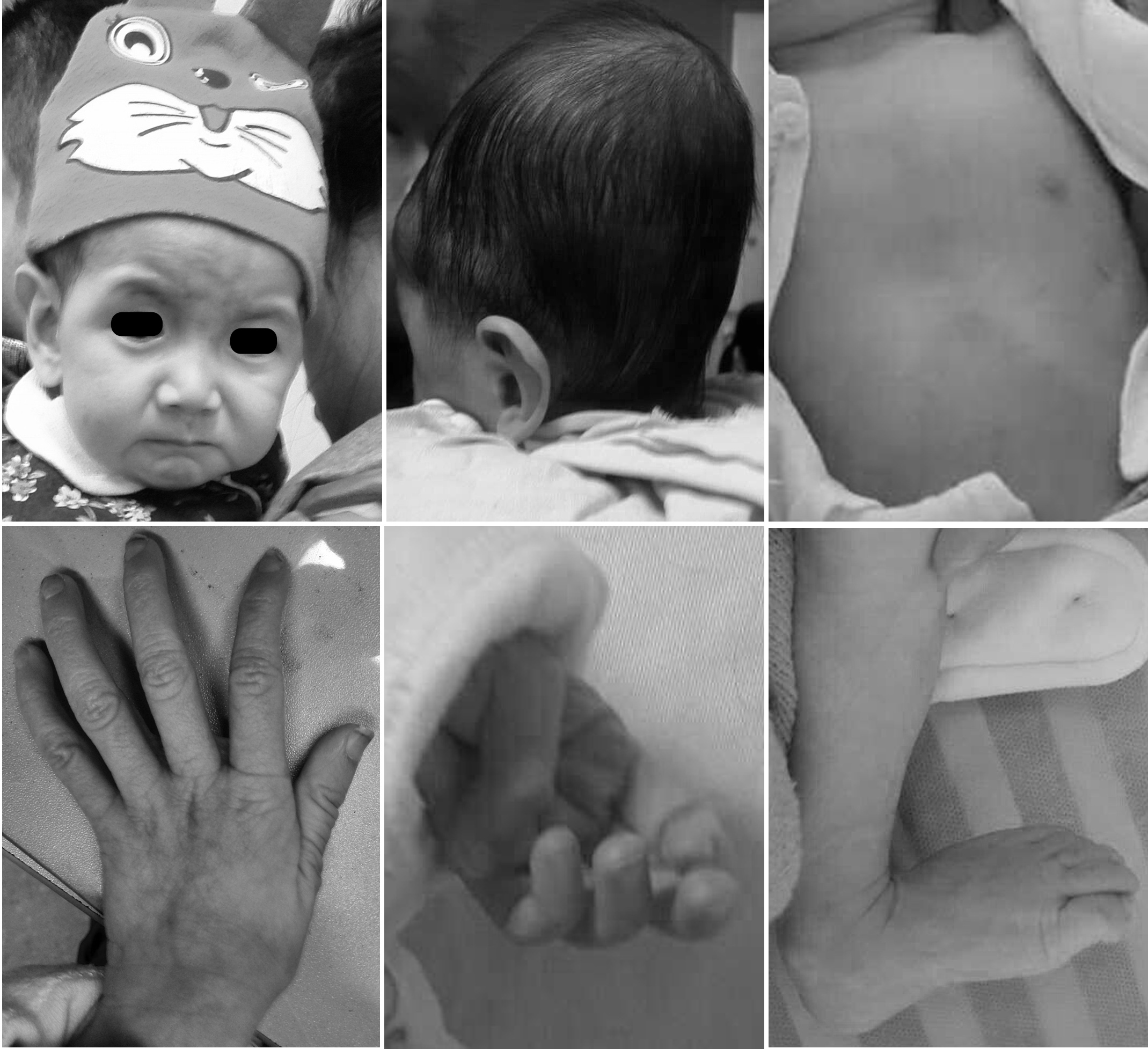

Supplement: Additional file 1: — Clinical features of the patient showing facial appearance, dolichocephaly, the pectus deformity, arachnodactyly, the thumb sign, and pes planus. (JPEG 896 kb) [file 12887_2016_598_MOESM1_ESM.jpeg]
